# Frontiers in Cell-Cycle-Targeting Therapies: Addressing the Heterogeneity of the Cancer Cell Cycle

**DOI:** 10.3390/cancers18020329

**Published:** 2026-01-21

**Authors:** Ishaar P. Ganesan, Hiroaki Kiyokawa

**Affiliations:** Department of Pharmacology, Feinberg School of Medicine, Northwestern University, Chicago, IL 60611, USA; ishaarganesan2024@u.northwestern.edu

**Keywords:** cyclin, CDK, retinoblastoma protein, checkpoint, WEE1, Aurora kinase, polo-like kinase, small molecules, cancer therapy

## Abstract

The cell division cycle machinery has been regarded as a promising therapeutic target for several decades. One of the most prominent milestones in the approach to targeting the cancer cell cycle was the development and approval of CDK4/6 inhibitors such as palbociclib, ribociclib, and abemaciclib. These small-molecule therapeutics have exhibited remarkable anti-cancer efficacy and have become primary choices for treating steroid receptor-positive breast cancer at multiple stages. This epoch-making success of cell-cycle-targeting drugs was followed by the development of small molecules to target other cell cycle-regulatory proteins, such as CDK2, CDK1, WEE1 kinase, Aurora kinases, and polo-like kinases, while therapeutic strategies to overcome resistance to CDK4/6 inhibitors have been pursued. In this article, we focus on heterogeneous vulnerabilities of cancers as consequences of various genetic and epigenetic alterations in the cell cycle-regulatory network, and we discuss how next-generation cell-cycle-targeting drugs currently in the developmental pipeline could exploit these heterogeneous vulnerabilities in the cancer cell cycle. We hope to provide a forward-looking perspective on directions for therapeutic cell-cycle targeting in the advent of personalized precision medicine.

## Simple Summary

While the fidelity of the physiological cell division cycle is maintained with multiple layers of fail-safe mechanisms, the cancer cell cycle differs from the normal cell cycle and is heterogeneous among cancers, with diverse genetic and epigenetic abnormalities that create vulnerabilities targetable by pharmacological agents. Here, we focus on the heterogeneity of cancer cell dependency on different CDKs and phase-specific cell cycle checkpoints, defined by key biomarkers; we then discuss how these molecular signatures could guide the rational use of small molecules targeting cell cycle-regulatory kinases in the developmental pipeline. This opinion is framed within the paradigm of personalized precision medicine.

## 1. Introduction

Cell-cycle-targeting drugs in the new generation are evolving rapidly, with several small-molecule inhibitors in development to address cancer-specific dysfunctions in the cell cycle machinery beyond the well-established target CDK4/6, including CDK2, CDK1, WEE1, Aurora kinases, and polo-like kinases (PLKs). These novel therapeutics aim to enhance specificity, overcome resistance, and address heterogeneity of cancers by selectively inhibiting key cell cycle-regulatory proteins that become vulnerabilities in various genetic and epigenetic contexts. Cancer heterogeneity is often described in multiple contexts, such as inter-tumoral heterogeneity, which highlights variations in genetic, epigenetic, and phenotypic properties, even in the same pathological subtypes of cancer, and intra-tumoral heterogeneity, describing the coexistence of distinct clonal populations of cancer cells in a tumor lesion or spatial organization of cancer stem cell niches and their differentiated progeny within the tumor. This intra-tumoral heterogeneity often extends to the tumor microenvironment, where multiple non-epithelial cell types, including immune cells, fibroblasts, and endothelial cells, exert diverse interactions with malignant cells. While numerous recent studies have indicated the significance of intra-tumoral heterogeneity, not only in the evolving process of tumor progression but also in the development of drug resistance, the topic has been extensively discussed in other excellent reviews [1,2,3,4,5]. In this opinion article, we focus on inter-tumoral heterogeneity, which is especially associated with cancer-specific alterations in the circuitry that coordinates the momentum and checkpoints of cell cycle progression, mainly through fine-tuning the activities of cyclin-dependent kinases (CDKs).

The cell cycle machinery has long been regarded as a primary anti-cancer therapeutic target. Conventional chemotherapeutic drugs that are still used clinically include S-phase-specific, DNA replication-dependent cytotoxic agents, e.g., cytosine arabinoside, 6-mercaptopurine, hydroxyurea, and methotrexate, and M-phase-specific microtubule toxins, e.g., vinca alkaloids and taxanes. The biggest issue associated with these conventional chemotherapies is clearly the adverse effects due to their nonselective cytotoxicity against physiologically proliferating tissues. During the last couple of decades, we have learned that this shortcoming of cell-cycle-targeting approaches may be overcome by the successful development and application of small-molecule inhibitors targeting CDKs and other cell cycle-regulatory kinases [6,7]. This notion is supported by observations that cancer cell-specific mechanisms of unrestricted cell cycle progression are heterogeneous, even within the same pathological subtypes, unlike the physiological cell division cycle protected by multiple layers of fail-safe mechanisms, as discussed below in Section 5. This inter-tumoral heterogeneity with diverse genetic and epigenetic abnormalities creates vulnerabilities, i.e., dependencies on specific gene(s) for survival, which can be targeted by small molecule-based targeted therapies. In this article, we will discuss small molecule-based therapies targeting various cell cycle-regulatory kinases from G_1_-regulatory CDK4 and CDK6 to other rate-limiting regulators of G_1_-S and G_2_-M transitions, including CDK2, CDK1, WEE1 kinase, Aurora kinases, and polo-like kinases (PLKs), together with approaches that exploit cell cycle checkpoints and DNA damage response liabilities [8,9,10]. These novel therapeutics aim to enhance anti-cancer selectivity and overcome resistance by targeting specific vulnerabilities.

## 2. Current Cell-Cycle-Targeting Therapies

CDK4/6 inhibitors—palbociclib, ribociclib, and abemaciclib—demonstrated excellent therapeutic efficacy in pivotal clinical trials for steroid receptor-positive, HER2-negative breast cancers, exemplified by the PALOMA-1, MONALEESA-2, MONALEESA-7, and MONARCH-E clinical trials [11,12,13,14,15]. These agents have collectively become the standard of care for this cancer type across disease stages [16,17,18]. The clinical success of these kinase inhibitors has definitively validated the cell cycle machinery as a prime therapeutic target in oncology, substantiating over three decades of preclinical evidence [6,19,20] and catalyzing the development and clinical evaluation of next-generation inhibitors targeting the broader spectrum of kinases governing cell cycle progression. Notably, recent clinical advancements, including the adjuvant approval of abemaciclib and ribociclib [14,15,21,22] and the emergence of combination regimens such as inavolisib–palbociclib–fulvestrant for PIK3CA-mutated tumors [23,24], underscore the shift toward decision-making processes guided by well-defined biomarkers and highlight the growing importance of patient stratification for achieving maximal therapeutic benefit.

## 3. Functional Redundancy Among Multiple CDKs and Lessons from Knockout Mouse Studies

**Figure 1:**
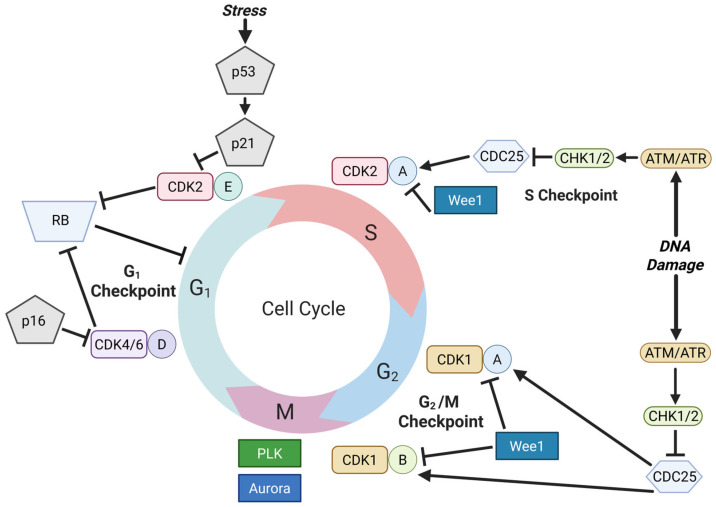
Mammalian cell cycle machinery and checkpoints. The colored segments of the circle denote cell cycle phases (G_1_, S, G_2_, M). Capital letters denote cyclins (D, E, A, and B); arrows indicate activation and bar-headed lines indicate inhibition.

It is established that progression of the mammalian cell cycle is regulated by several cyclin-CDK complexes in a temporal manner (Figure 1) [25]. Three D-type cyclins (D1, D2, and D3), which are expressed differentially in cell type-specific fashions, form complexes with either CDK4 or CDK6. These cyclin D-CDK4/6 complexes play a critical role in initiating phosphorylation of the retinoblastoma tumor suppressor protein (RB) at several phosphorylation sites during mid-G_1_ phase and gradually activate the E2F transcription factors by counteracting RB-mediated repressor activity. Subsequently, cyclin E expression accumulates in an E2F-dependent manner, and the cyclin E-CDK2 complex becomes fully active just prior to the G_1_-S transition, thereby completing phosphorylation of RB at up to 16 sites and leading to maximum transactivation of the E2F target genes required for S phase. This coordinated control of the RB-E2F activity by the G_1_ CDKs forms the dynamic core of the G_1_ checkpoint, governing the duration of the G_1_ phase adjusted for cell fate-determination processes. It should be noted that the cyclin E-CDK2 complex also plays RB-E2F-independent roles in the G_1_-S transition, by direct phosphorylation of components of the pre-replication complex, including CDC6 and RECQL4, as well as by phosphorylation of transcription factors such as MYC and FOXO [26,27,28]. Moreover, cyclin E-CDK2 plays a key role in downregulating the G_1_-specific ubiquitin ligase APC/C-CDH1, to terminate the proteolysis-dominant G_1_ phase and allow accumulation of proteins required for S-phase, i.e., cyclins E and A and replication factors [29]. Specifically, under stressful conditions during G_1_, activated p53 induces the CDK inhibitor p21, causing G_1_-S arrest mainly by CDK2 inhibition [30,31,32]. Following the G_1_-S transition, the cyclin A-CDK2 activity functions as the central momentum of S phase progression and becomes the major target of the DNA damage-induced S phase checkpoint. In response to replication stress or DNA damage, ATR and ATM signal through CHK1/CHK2 to inhibit CDC25 phosphatases and restrain CDK2 activity, inducing an intra-S-phase slowdown. This mechanism delays progression into G_2_ while repair is engaged, providing a rationale for therapies targeting such DNA repair-related vulnerabilities in genetically defined contexts [33,34]. For the G_2_-M transition, CDK1 plays a dominant role in complexes with cyclins A and B, and the timing of CDK1 activation is controlled precisely by the G_2_-M checkpoint, which involves inhibitory phosphorylation of CDK1 at Tyr15 catalyzed by the WEE1 kinase. DNA damage during G_2_ blocks the G_2_-M transition mainly by the ATM/ATR-CHK1/2-CDC25-CDK1 checkpoint axis, preventing the damaged cell from undergoing mitosis and cytokinesis with potentially malignant genetic lesions. Upon the release of CDK1 from the inhibition mediated by WEE1, the cyclin A- and cyclin B-CDK1 complexes become active and collaborate with the mitosis-specific kinases, such as Aurora kinase and PLKs, to control the progression of mitosis.

Phenotypes of CDK knockout mice suggest that the G_1_ CDK genes, such as *Cdk4*, *Cdk6*, and *Cdk2*, play partially overlapping but still distinct roles in mammalian development and tissue homeostasis. Mice with germline disruption of the *Cdk4*, *Cdk6*, or *Cdk2* gene survive embryogenesis and are viable, while adult mutant mice exhibit diverse phenotypes in tissue-specific manners [35,36,37,38,39]. Analyses of compound knockout mice such as *Cdk4*/*Cdk2*, *Cdk4*/*Cdk6*, or *Cdk6*/*Cdk2* double-null mice have demonstrated exacerbated phenotypes relative to single-knockout mice, suggesting that these G_1_ CDKs play overlapping roles in development and tissue homeostasis [39,40,41,42]. Importantly, cell cycle progression of cultured primary cells, e.g., embryonic fibroblasts, may continue even without these three CDKs, as long as CDK1 expression is intact [41]. The phenotypes of the experimental knockout models imply that physiological organs are equipped with fail-proof mechanisms to maintain proliferation and homeostasis without relying on a single CDK. This notion is consistent with the clinical observations that most adult cancer patients tolerate the clinically available CDK4/6 inhibitors and CDK2 inhibitors in clinical trials. In contrast to the compensatory regulation of the physiological cell cycle by multiple G_1_ CDKs, the cancer cell cycle seems to be more heterogeneous in nature and is often dependent on a single CDK, as previous studies demonstrated that *Cdk4*-null mice or *Cdk2*-null mice are resistant to tumorigenesis upon specific oncogene activation, e.g., *Neu*, *Ras*, or *Myc* [43,44,45,46,47]. These studies, together with numerous ex vivo experiments, have provided the scientific foundation for the therapeutic targeting of the cancer cell cycle machinery.

## 4. New-Generation Drugs in Development

The developmental pipeline now includes small-molecule inhibitors for CDK2, CDK1, WEE1, Aurora kinases, and PLKs, as well as novel strategies such as CDK degraders and highly selective agents intended to minimize toxicity. These efforts are supported by numerous ongoing preclinical and clinical trials [48,49], driven by a need to overcome resistance mechanisms and fine-tune therapeutic responses. Cell-cycle-targeting drugs for oncology make up a significant portion of efforts for developmental therapeutics ongoing in 2025, with many candidates designed for precision use in tumors with defined molecular lesions [50].

Within this developing paradigm, recent pharmacologic studies have clarified dosing and combination strategies, informing the advent of highly selective CDK2 inhibitors [50,51,52]. Multiple ATP-competitive CDK2 inhibitors, including INX-315 (NCT05735080) [53], INCB123667 (NCT07023627; NCT05238922; NCT07214779) [50], and PF-07104091 (NCT04553133; NCT05262400) [54,55] have progressed into early-phase clinical evaluation in biomarker-enriched cohorts, with INCB123667 advancing to Phase 3 development. These trials predominantly enroll patients with hormone receptor-positive breast cancer previously treated with CDK4/6 inhibitors, high-grade serous ovarian cancer, uterine serous carcinoma, and other solid tumors characterized by cyclin E overexpression or *CCNE1* amplification [50]. A current major challenge for the CDK2 inhibitors is dosage limitations driven by on-target myelosuppression, as demonstrated in early-phase trials [55]. Another challenge is drug resistance to the CDK2 inhibitors, often mediated by adaptive cyclin E overexpression [56]. However, this issue may be overcome by rational drug design, as PROTAC degraders targeting CDK2, such as NKT3964 (NCT06586957), demonstrated prolonged CDK2 depletion without cyclin E accumulation [57,58,59]. The success of the CDK2 targeting strategy will require further addressing dosage limitations, optimizing the mode of molecular inhibition and delivery, and evaluating combination regimens involving CDK4/6 inhibitors or DNA damage response modulators for robust anti-cancer efficacy.

It is noteworthy that among the actively pursued therapeutic targets, CDK1 attracts renewed attention. As the non-redundant CDK required for mitotic progression, CDK1 has emerged as a potential vulnerability in cancers with specific genetic backgrounds, particularly RB-deficient and triple-negative breast cancers [60,61,62,63]. Despite longstanding challenges in developing inhibitors that can distinguish CDK1 activity from that of CDK2, recent advances in medicinal chemistry have yielded agents with robust selectivity for CDK1 and appreciable preclinical efficacy [64]. Such CDK1-selective inhibitors may be able to target mitotic dependency in RB-deficient cancer types if proper therapeutic windows are identified.

The therapeutic exploitation of mitosis-specific kinases is further exemplified by advances in targeting Aurora kinase and PLK [65]. Aurora kinase A inhibitors (alisertib, LY3295668) (NCT04555837; NCT04106219; NCT06095505) [66,67,68,69,70] and Aurora kinase B inhibitors (AZD1152/AZD2811) (NCT03366675; NCT04525391; NCT04745689) [71,72,73] have demonstrated the ability to trigger mitotic catastrophe, particularly in cancers characterized by chromosomal instability or RB-deficiency, such as small-cell lung cancer and aggressive breast cancer subtypes. The development of PLK1 inhibitors, including volasertib (NCT05450965) [74,75,76], reflects a concurrent pursuit against aberrant mitotic progression in tumor cells; however, clinical progress has been tempered by nonselective toxicity, underscoring the centrality of precision medicine in future efforts.

## 5. Addressing Heterogeneity in Cancer Cell Cycle

**Figure 2:**
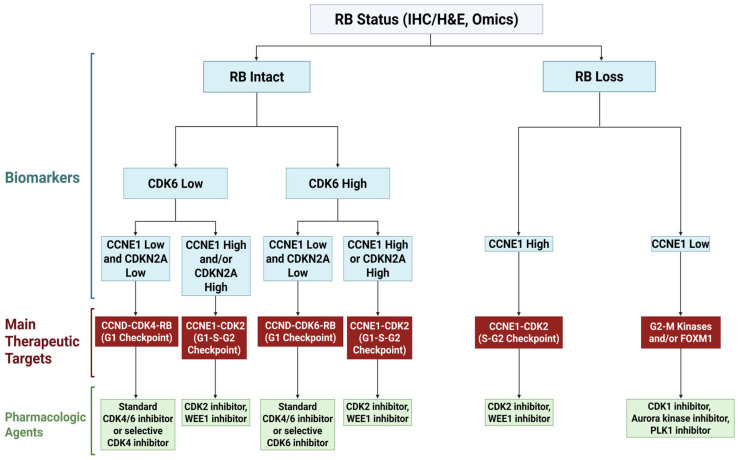
An example of rational biomarker-based choices of cell-cycle-targeting therapeutics.

A current mainstream direction in cancer therapeutics is the personalized approach to targeting specific vulnerabilities of individual cancers, which are consequences of cumulative genetic and epigenetic abnormalities during oncogenesis. Oncogenic alterations in cell cycle-regulatory genes are likely to transform the cell cycle machinery from the physiological state equipped with layers of fail-safe mechanisms to the cancer-specific state that may depend on a single prominent cell cycle driver, e.g., cyclin D-CDK4. These dependency states may be shaped not only by lesions within the cyclin-CDK complexes themselves, but also by changes in upstream mitogenic signaling pathways that converge on cyclin D expression (e.g., *RAS* or *WNT* activation) or cyclin E expression (e.g., *FBXW7* mutations). Cancer-specific vulnerabilities may also involve genes that are part of the cell cycle checkpoint and repair circuitry (see Section 2) [77,78,79]. It is noteworthy that dependency on the cell cycle checkpoint pathways is often amplified in tumors with impaired homologous recombination repair (e.g., *BRCA1/2*-altered contexts) [80,81,82]. Importantly, cancer cell-specific dependency or the “addiction” state is heterogeneous among individual cancers with different genetic and epigenetic alterations. For example, the steroid receptor-positive, HER2-negative subgroup of breast cancers exhibits strong CDK4 dependency and robust sensitivity to CDK4/6 inhibitors, whereas the triple-negative subgroup of breast cancers rarely responds to the CDK4/6-targeting therapies [83]. Molecular profiling and identification of reliable biomarkers are pivotal in matching advanced agents, either as monotherapies or combination therapies, to patient-specific cancer vulnerabilities, promising improved efficacy and reduced side effects [84,85]. For optimal applications of cell-cycle-targeting therapies, experimental efforts have been made to reveal genetic contexts that determine the efficacy of the CDK4/6 inhibitors and other cell-cycle-targeting agents in the developmental pipeline [86,87,88]. Among numerous studies focused on genetic profiling and pharmacological responsiveness, seminal investigations regarding cell-cycle targeting took advantage of the Cancer Dependency Map (DepMap), which is a collaborative research database of genetic vulnerabilities from CRISPR- and RNAi-based screens and provides a comprehensive map of genes that are required for proliferation and viability of more than 1200 cancer cell lines [89]. Data on cancer cell dependencies in DepMap have been extensively analyzed in comparison with data from genomics, next-generation sequencing, and cellular responsiveness to pharmacological agents, leading to the identification of novel genetic categories that predict clinical responses to specific anti-cancer agents, including the CDK4/6 inhibitors [90,91,92]. Based on the information from these linkage analyses, an example of stratified biomarker-guided applications of cell-cycle-targeting drugs for personalized medicine is shown in Figure 2.

The most prominent genetic alteration that determines the sensitivity of individual cancers to the standard CDK4/6 inhibitors is the status of the *RB1* gene-encoding RB. The loss of RB function due to *RB1* deletions/mutations or inactivation by viral oncoproteins marks cell-intrinsic independence from CDK4 and CDK6 for proliferation, and RB loss has become the most reliable biomarker for resistance to the CDK4/6 inhibitors [87,88,90,92,93,94,95]. The integrated genomic approach further suggests that the following four categories apply to the *RB1*-intact group of cancers: CDK4-selective dependence, CDK6-selective dependence, dual CDK4/6 dependence, and CDK4/6 independence. CDK6 expression is a dominant predictive biomarker that correlates positively with CDK6 dependence and inversely with CDK4 dependence in the genetic linkage studies [90]. In addition, tissue lineage-specific patterns may provide useful clinical guidance. Adenocarcinomas tend to demonstrate preferential CDK4 dependence, whereas hematologic malignancies and squamous cell carcinomas show greater CDK6 dependence. These categories are expected to better guide clinical decisions when CDK4-selective and CDK6-selective inhibitors become available in the future [86,90,92].

In addition to the categories based on CDK4- and CDK6-dependence, RB-proficient tumors may be further categorized according to two biomarkers, such as cyclin E encoded by the *CCNE1* gene and p16 encoded by the *CDKN2A* gene. In a particular subgroup of cancers with representative cyclin E/*CCNE1* overexpression, the RB-dependent G_1_ checkpoint is compromised, possibly leading to an uncoupled transition from G_1_ to S, solely dependent on the cyclin E-CDK2 activity. Overexpression of p16/*CDKN2A*, an intrinsic CDK4/6 inhibitor, is another reliable indicator of cancer independence from the cyclin D-CDK4/6 pathway [86,87,90,91,92,93,96]. This regulatory shift toward the cyclin E-CDK2 pathway creates distinct therapeutic vulnerabilities, including cellular dependence on the S phase licensing and checkpoint machinery, as well as on the G_2_ checkpoint. Thus, *CCNE1* and *CDKN2A* are clinically tractable biomarkers of the “CDK2-addictive” state. Tumors with this signature would not respond to the CDK4/6 inhibitors and represent good candidates for CDK2-targeting therapies [90,91,92,93,96]. Treating CDK2-addictive, RB-intact cancers with CDK2 inhibitors has been shown to cause a G_1_ arrest characterized by RB dephosphorylation and E2F suppression. However, at higher doses, CDK2 inhibitors stall cell cycle progression in G_2_ with increased WEE1-dependent phosphorylation of CDK1 at Tyr15 and elevated cyclin B1 levels [97]. This phenotype suggests sustained activation of the S and G_2_ checkpoints and offers opportunities for combination therapies with agents targeting these checkpoints required for survival of CDK2-addictive tumors, such as inhibitors targeting the aforementioned DNA damage-responsive ATM/ATR-CHK1-CHK2 pathways and WEE1 inhibitors [91,92,93,98,99]. In RB-deficient cancer cells without the function of the G_1_ checkpoint, the G_2_ checkpoint, along with the mitosis machinery, is likely to form vulnerabilities. WEE1 inhibitors and antagonists of mitotic kinases, i.e., CDK1, Aurora kinases, and PLKs, would represent rational therapeutic strategies for such cancers [48,86,90,92,93,94,98]. Another potential approach to target the mitosis machinery is the pharmacological inhibition of FOXM1, a master transcription factor for the mitotic kinases and regulators [100]. Several FOXM1 inhibitors have been developed and tested in preclinical models [101,102,103]. CDK2 inhibitors would still be a choice for treating RB-deficient cancers, especially when *CCNE1* overexpression is observed [90,91,92].

Taken together, the datasets from the integrated functional genomics studies provide a scientific framework for the identification of more reliable biomarkers for next-generation cell-cycle-targeting drugs and their prospective clinical applications.

## 6. Conclusions

Personalized precision medicine is central to future cell-cycle-targeting therapy, enabling rational development of tailored plans not only for initial therapies but also for tackling drug resistance, based on molecular characterizations of surgical samples, biopsy specimens, or circulating tumor cells using next-generation sequencing. Prospective validation of biomarkers beyond *RB1*, *CDK6*, *CCNE1*, and *CDKN2A* will drive a patient stratification that, when integrated with functional genomic datasets, will be essential for practical clinical decision-making maps. Moreover, temporal or adaptive changes in individual cancer signatures associated with clinical evolution, i.e., further tumor progression and treatment response, should be identified and addressed by rational combination strategies to prevent and overcome drug resistance. In parallel, as exemplified by the clinical trials of CDK2, CDK1, and mitotic kinase inhibitors facing challenges associated with adverse effects, it is crucial for us to define the proper therapeutic windows for cell-cycle-targeting drugs. We expect that the individualized approach based on functional genomics and biomarker assessments will help discern those therapeutic windows, because furthering our knowledge about the dependence of individual cancers on specific regulatory nodes would enable a rational choice of treatment dosage and duration, as well as combination schemes. In addition, resistance-aware designs for future drugs are expected to optimize treatment intensity over time, limiting cumulative toxicity while preserving efficacy. Beyond such stratification addressing the heterogeneous cancer cell cycle, efforts for complementary innovation are ongoing to address intra-tumoral heterogeneity, e.g., selective control of spatially confined resistant compartments [2,3,104]. Collectively, these therapeutic frameworks for addressing cancer heterogeneity shift cell-cycle targeting from indiscriminate maximal inhibition toward context- and time-resolved therapeutic delivery, offering a rational path toward safer, more durable, and more personalized cancer treatment for precision medicine.
